# Combined Application of High-Throughput Sequencing and Metabolomics Reveals Metabolically Active Microorganisms During Panxian Ham Processing

**DOI:** 10.3389/fmicb.2019.03012

**Published:** 2020-01-10

**Authors:** Yu Mu, Wei Su, Yingchun Mu, Li Jiang

**Affiliations:** ^1^School of Liquor and Food Engineering, Guizhou University, Guiyang, China; ^2^Guizhou Provincial Key Laboratory of Agricultural and Animal Products Storage and Processing, Guizhou University, Guiyang, China

**Keywords:** Panxian ham, metabolomics, high-throughput sequencing, non-volatile metabolites, metabolically active microorganism

## Abstract

Panxian ham, a traditional Chinese dry-cured ham, is protected by national geographical indication. Similar to other fermented foods, the microbial population of dry-cured ham is pivotal to taste and flavor formation. This study aimed to establish the relationship between microorganisms and metabolites during the spontaneous fermentation of Panxian ham. Multivariate analysis based on metabolomics data revealed that continuous metabolic changes occurred during the entire fermentation process, with the most significant changes occurring in the initial stage of ripening. Thirty-one significantly different metabolites (SDMs) were identified as discriminant factor, and pathway analysis suggested that these metabolites were involved in 30 pathways, including alanine, aspartate, and glutamate metabolism; glycine, serine, and threonine metabolism; and arginine and proline metabolism. Microbial community analysis using the Illumina MiSeq platform indicated that the bacterial community was more complex than the fungal community, and their succession regulation differed during processing. At the genus level, 11 bacteria and five fungi were identified as core microbes, of which *Staphylococcus* was the dominant bacteria and *Debaryomyces* and *Aspergillus* were the dominant fungi. Further, statistical redundancy analysis (RDA) indicated that *Staphylococcus*, *Debaryomyces*, and *Chromohalobacter* promoted the production of amino and fatty acids; *Cobetia* and *Aspergillus* were associated with sugar metabolism, and *Kushneria*, *Penicillium*, and *Yamadazyma* were closely related with organic acids. These findings provide fundamental knowledge regarding the metabolically active microorganisms in Panxian ham, helping industrial processors to develop effective strategies for standardizing quality parameters.

## Introduction

Dry-cured ham is a traditional fermented meat product with a long history of consumption that is very popular around the world for its unique sensory properties and high nutritive value. Previous studies have suggested that dry-cured ham may be included as a conventional part of a healthy diet (Fernández et al., 2007; Jimenez-Colmenero et al., 2010), because many nutritional and functional substances are accumulated during fermentation, such as the essential amino acids, taurine, cysteine, carnosine, creatinine, anserine, and glutathione (Marusic et al., 2013). In addition, dry-cured ham is also a valuable source of bioactive peptides. Recent reports indicate that regular consumption of dry-cured ham can provide protective health benefits, such as reducing the risk of cardiovascular disease and inhibiting the activation of platelets and monocytes (Martinez-Sanchez et al., 2017; Montoro-Garcia et al., 2017).

Panxian ham, one of the famous dry-cured hams in China, is mainly distributed in Guizhou and its neighboring provinces, and is protected as a national geographical indication product (following Jinhua and Xuanwei hams) due to its strong characteristic taste, flavor, aroma, and texture; which are attributable to the Panxian region. Similar to most dry-cured hams, the production of Panxian ham usually consists of raw material selection, salting, resting, and drying-ripening. The entire process is usually accompanied by a series of complicated biochemical and enzymatic reactions, such as protein degradation, lipid oxidation, glycolysis, and Maillard reactions (Zhou and Zhao, 2007; Petrova et al., 2015). It is well known that endogenous enzymes play essential in these reactions (Toldrá and Flores, 2000), but the enzymatic action of microorganisms cannot be discounted (Simoncini et al., 2007; Landeta et al., 2013). The diversity of fermented meat’s microbial community could be influenced by geographical location and production environment due to the traditional spontaneous fermentation that occurs during processing (Van Reckem et al., 2019). This is especially important because microbial diversity has the potential to affect the metabolome, and thus the quality and typicity of fermented foods (Montanari et al., 2018). However, previous studies on dry-cured ham have focused on the relationship between microorganisms and volatile flavors (Ge et al., 2017; Mu et al., 2019), while the effect of microorganisms on non-volatile metabolites has not yet been studied.

In recent years, multivariate analysis of microorganisms and metabolites has been applied to improving the quality of fermented foods (Park et al., 2019; Wurihan et al., 2019). Thus, exploration of microbial community succession, metabolic changes, and their interrelationship during processing, is warranted to improve Panxian ham quality. Previous studies, based on culture-dependent methods, have investigated changes in microbial diversity during ham production (Tu et al., 2010). In fact, the majority of microorganisms cannot be detected by culture-dependent methods due to their “viable but non-culturable” state (Fakruddin et al., 2013). High-throughput sequencing (HTS) technology allows a deeper and more precise evaluation of complex microbiota with lower cost and shorter analysis time (Tamang et al., 2016), and has successfully characterized microbial community succession during the spontaneous fermentation of sausage (Wang et al., 2019). Moreover, nuclear magnetic resonance (NMR)-based metabolomics analysis has revealed metabolic changes during dry-cured ham processing (Zhang et al., 2019), but has lower sensitivity for detecting low abundance metabolites compared with the MS-based technique (Sugimoto et al., 2017).

Indigenous microorganisms are known to play a pivotal role in the final taste, flavor, and texture of fermented foods. However, little information is available about the functional microorganisms in Panxian ham; this frequently leads to fluctuations in quality among different batches. Hence, a gas chromatography time of flight mass spectrometry (GC-TOF-MS)-based metabolomics approach and HTS technology were applied to monitor the evolution of the Panxian ham metabolite profile and microbial community structure during processing. Furthermore, multivariate redundancy analysis (RDA) was conducted to uncover the correlation between dominant microbes and significantly different metabolites (SDMs). It is hoped that this study will aid in the standardization of Panxian ham production throughout the region.

## Materials and Methods

### Processing of Dry-Cured Hams

Thirty fresh pork thighs weighing between 10–12 kg from a local slaughterhouse were selected and processed in a Panxian ham food factory (Panzhou, China) according to traditional methods, including aging, salting, resting, and ripening. The raw hams were aged for 24 h and then salted with sea salt in 4°C cold storage. The curing process occurred in three stages over 21 days; the first use of salt was 4% of the weight of the thigh, and the second and third uses were 2% each, for a total amount of 8% salt based on the weight of the thigh. After salting, the salt and blood remaining on the surface of the ham was rinsed. Then, the hams were transferred to a room for 30 days of rest at 10–15°C and 75–80% relative humidity (RH). Finally, the hams were hung in a fermentation room at 20–22°C and 65–70% RH until fully ripened.

### Sampling

The biceps femoris muscles were collected at six different stages of ham processing: raw ham, post-salting (21 days), post-resting (51 days), and initial stage (180 days), middle stage (360 days), and final stage (540 days) of ripening. Five hams were sampled randomly at each stage during processing for parallel testing. After removal of subcutaneous fat and connective tissue, the samples were vacuum-packaged and stored at −80°C until analysis.

### Metabolites Extraction and GC-TOF-MS Analysis

Ham sample (300 ± 1 mg) was placed in the 5 mL EP tubes with 3 mL extraction liquid (V_*Methanol*_: *V*_*Chloroform*_ = 3:1), then 5 μL of L-2-Chlorophenylalanine (1 mg/mL stock in dH_2_O) was added as internal standard followed by vortex-mixing for 30 s. The mixture was homogenized in a ball mill for 4 min at 45 Hz and treated with ultrasound for 5 min (incubated in ice water). Next, the centrifugal process was conducted at 12,000 rpm and 4°C for 15 min, and 50 μL of sample supernatant was transferred into a fresh 1.5 mL EP tubes. In addition, quality control (QC) samples were prepared by pooling 20 μL of supernatant from each sample. Then the supernatant was completely dried in a vacuum concentrator (without heating) and 80 μL of methoxyamination hydrochloride in pyridine solution (20 mg/mL) was added and incubated for 30 min at 80°C. Then derivatized using 40 μL of the BSTFA (Bistrifluoroacetamide) [1% TMCS (Templated mesoporous carbons), v/v] at 70°C for 1.5 h. Finally, 5 μL of FAMEs (Fatty acid methyl esters) in chloroform was added to the QC sample when cooling to the room temperature. All samples were analyzed by gas chromatograph system coupled with a Pegasus HT time-of-flight mass spectrometer (GC-TOF-MS).

GC-TOF-MS analysis was performed using an Agilent 7890 gas chromatograph system coupled with a Pegasus HT time-of-flight mass spectrometer. The system utilized a DB-5MS capillary column coated with 5% diphenyl cross-linked with 95% dimethylpolysiloxane (30 m × 250 μm inner diameter, 0.25 μm film thickness; J&W Scientific, Folsom, CA, United States). A 1 μL aliquot of the analyte was injected in splitless mode. Helium was used as the carrier gas, the front inlet purge flow was 3 mL/min, and the gas flow rate through the column was 1 mL/min. The initial temperature was kept at 50°C for 1 min, then raised to 310°C at a rate of 10°C/min, then kept for 8 min at 310°C. The injection, transfer line, and ion source temperatures were 280, 280, and 250°C, respectively. The energy was −70 eV in electron impact mode. The mass spectrometry data were acquired in full-scan mode with the *m*/*z* range of 50–500 at a rate of 12.5 spectra per second after a solvent delay of 6.33 min.

### Metabolome Data Processing and Analysis

Chroma TOF 4.3X software of LECO Corporation and LECO-Fiehn Rtx5 database were used for raw peaks exacting, the data baselines filtering and calibration of the baseline, peak alignment, deconvolution analysis, peak identification and integration of the peak area (Kind et al., 2009). Both of mass spectrum match and retention index match were considered in metabolites identification. Remove peaks detected in <50% of QC samples or RSD > 30% in QC samples (Dunn et al., 2011). The data including the compound name, peak area, retention time, similarity to metabolites in the database, and mass (*m*/*z*) were ultimately imported into Microsoft Excel.

The compound name and peak area information were selected as variables for multivariate statistical analysis using SIMCA-P 14.1 (Umetrics, Umea, Sweden). The GC-TOF-MS data sets were visualized with unsupervised principal component analysis (PCA) and supervised orthogonal partial least squares discriminant analysis (OPLS-DA). Meanwhile, the cross-validation analysis of variance (CV-ANOVA) was performed to evaluate the OPLS-DA model. Based on OPLS-DA model and one-way analysis of variance (ANOVA), the variable importance in projection (VIP) values and *p*-value were calculated and the SDMs were determined. The peak area of SDMs was visualized via R software (v3.6.1) with the “pheatmap” package after log-transformation. Pathway and enrichment analyses were conducted by MetaboAnalyst 4.0^1^, which is a free and web-based tool with a high-quality KEGG (Kyoto encyclopedia of genes and genomes) metabolic pathway.

### DNA Extraction and PCR Amplification

Total bacterial DNA were extracted from samples using the Power Soil DNA Isolation Kit (MO BIO Laboratories) according to the manufacturer’s protocol. DNA quality and quantity were assessed by the ratios of 260/280 nm and 260/230 nm. Then DNA was stored at −80°C until further processing. The V3-V4 region of the bacteria 16S rRNA gene was amplified with primers 338F (5′-ACTCCTACGGGAGGCAGCAG-3′) and 806R (5′-GGACTACHVGGGTWTCTAAT-3′). The ITS1 region of the fungi was amplified with the forward primer ITS1F (5′-CTTGGTCATTTAGAGGAAGTAA-3′) and the reverse primer ITS1R (5′-GCTGCGTTCTTCATCGATGC-3′).

PCR amplification was performed in a total volume of 50 μL, which contained 10 μL Buffer, 0.2 μL Q5 High-Fidelity DNA Polymerase, 10 μL High GC Enhancer, 1 μL dNTP, 10 μM of each primer and 60 ng genome DNA. Thermal cycling conditions were as follows: an initial denaturation at 95°C for 5 min, followed by 15 cycles at 95°C for 1 min, 50°C for 1 min and 72°C for 1 min, with a final extension at 72°C for 7 min. The PCR products from the first step PCR were purified through VAHTSTM DNA Clean Beads. A second round PCR was then performed in a 40 μL reaction which contained 20 μL 2 × Phusion HF MM, 8 μL ddH_2_O, 10 μM of each primer and 10 μL PCR products from the first step. Thermal cycling conditions were as follows: an initial denaturation at 98°C for 30 s, followed by 10 cycles at 98°C for 10 s, 65°C for 30 s and 72°C for 30 s, with a final extension at 72°C for 5 min. Finally, all PCR products were quantified by Nanodrop 2000 and pooled together. HTS analysis of bacterial rRNA and fungal ITS1 genes was performed on the purified, pooled sample using the Illumina Hiseq 2500 platform (2 × 250 paired ends).

### Microbiome Data Processing and Bioinformatics Analysis

Raw fastq files were demultiplexed, quality-filtered by Trimmomatic and merged by FLASH (Magoc and Salzberg, 2011). Operational taxonomic units (OTUs) were clustered with 97% similarity cutoff using UPARSE (Bokulich et al., 2013). Then, the taxonomy of each sequence was analyzed by Ribosomal Database Project (RDP) Classifier against the Silva (SSU123) database using confidence threshold of 80% (Fadrosh et al., 2014). Finally, chimeric sequences were identified and removed using UCHIME. The alpha diversity indices, rarefaction curves, and the number of unique OTUs in each sample were evaluated using the Mothur software (Kozich et al., 2013). For beta diversity analysis, principal coordinate analysis (PCoA) and non-metric multidimensional scaling (NMDS) were performed using QIIME software (Caporaso et al., 2010). Prediction of gene function was performed using PICRUSt (phylogenetic investigation of communities by reconstruction of unobserved states) (Langille et al., 2013). To establish the co-occurrence network between core bacterial and fungal genus, we used R software with the “corrplot” package for calculating Pearson’s correlation coefficient and applied Cytoscape software for visualization.

### Integrative Analysis of Metabolome and Microbiome Data

The relative abundance of core microbes (≥1%) and the relative content of SDMs (VIP > 1 and *p* < 0.05) were used for the integrative analysis. The multivariate RDA based on the “vegan” package was performed by R software.

### Statistical Analysis

Statistical analysis was performed using SPSS 22.0 software. The data obtained were subjected to one-way analysis of variance (ANOVA), and the significance level in the analyses was considered at *p* < 0.05.

## Results and Discussion

### Metabolite Profile Comparison of Ham Samples at Different Stages

**FIGURE 1:**
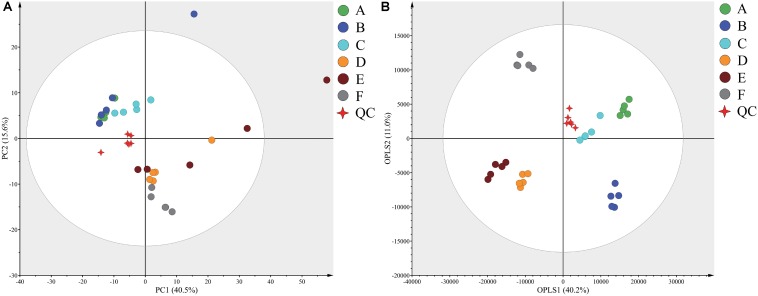
Principal component analysis (PCA) **(A)** and orthogonal partial least squares discriminant analysis (OPLS-DA) **(B)** score plots derived from the GC-TOF-MS data from Panxian ham and Quality Control (QC) samples. A–F indicate the traditional spontaneous fermentation process stage of Panxian ham: *(A)* raw ham; *(B)* post-salting; *(C)* post-resting; *(D)* initial stage of ripening; *(E)* middle stage of ripening; *(F)* final stage of ripening. *(QC)* represents the quality control sample.

Typical GC-TOF-MS total ion chromatograms (TICs) of dry-cured ham samples from the six different stages are shown in Supplementary Figure S1. In total, 578 signal features were identified in all samples and 527 remained after applying an imputation model with an 80% limit for missing data. PCA of the entire set of measured analytes was used to investigate gross changes in metabolic physiology of Panxian ham during processing. The PCA score plot showed 40.5% and 15.6% variances by PC1 and PC2, respectively (Figure 1A). As fermentation progressed, a typical metabolite drift was observed. Specifically, metabolite profiles were distributed along PC1 and progressed from quadrants 2 to 4, which illustrated that continuous metabolic changes occurred during processing. In the PCA score plot, a clear separation between post-resting and initial stage of ripening was found, indicating that extremely rapid metabolite changes transpired in this period. To maximize the distinction between different samples, and to obtain a better understanding of the metabolites responsible for the separation, the OPLS-DA model was applied. Compared with the PCA model, the *R*^2^ and *Q*^2^ values for evaluation of model quality increased, and all samples in the OPLS-DA score plot were within the 95% confidence interval (Figure 1B). Meanwhile, the CV-ANOVA *p*-value for the OPLS-DA model generated from the original data matrix was 1.67 × 10^–22^, indicating this model’s improved predictive ability (Bradley and Robert, 2016).

### Identification of Significantly Different Metabolites and Analysis of Metabolic Pathways During Processing

**FIGURE 2:**
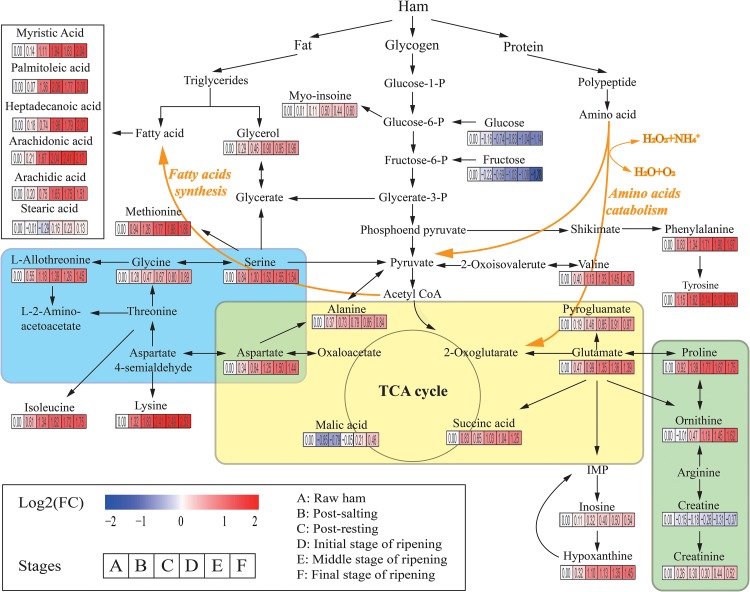
The integrated metabolic pathway shows the dynamics of significantly different metabolites (SDMs) during Panxian ham processing. Beneath each metabolite, the color gradient and their values indicate the log2 (fold change) with respect to raw ham: red and blue represent up and downregulation of metabolites, respectively. Glycine, serine, and threonine metabolism, alanine, aspartate, and glutamate metabolism, and arginine and proline metabolism are shown in the blue, yellow, and green areas, respectively.

A total of 226 metabolites were identified by mass spectrum matching and 80 reliable metabolites were determined based on spectral similarity values >700 (Hao et al., 2019). The differential variables among groups were selected based on VIP scores >1.0 from OPLS-DA and *p* < 0.05 from ANOVA. In total, 31 SDMs were identified, including 15 amino acids, 6 fatty acids, 3 organic acids, 2 sugars, 2 polyols, 2 nucleic acids, and 1 other metabolite (Supplementary Table S1). To identify the most relevant metabolic pathways during Panxian ham processing, all SDMs were evaluated using metabolic pathway and enrichment analysis with MetaboAnalyst 4.0. Thirty pathways were obtained and “alanine, aspartate, and glutamate metabolism,” “glycine, serine, and threonine metabolism,” and “arginine and proline metabolism” were determined as the most relevant metabolic pathways (Supplementary Figure S2). Figure 2 shows the integrated metabolic pathway adapted from the KEGG database^2^ and previous studies (Toldrá and Flores, 2000), including protein, fat, and glycogen metabolism.

Amino acids, often the most important primary metabolites in dry-cured ham because of the high protein content of pork thigh, had a crucial role on the formation of taste in dry-cured hams (Zhang et al., 2018; Liu et al., 2019). Specifically, alanine, valine, and serine contributed to perceived sweetness, glutamate, pyroglutamate, and aspartate were responsible for the umami taste, while tyrosine and lysine were associated with the unique aged taste (Careri et al., 1993; Zheng et al., 2015). In addition, amino acids were the precursors of many volatile flavors and played an indispensable role in the flavor formation of dry-cured ham (Shi et al., 2019). In the present study, the levels of all amino acids increased rapidly until the initial stage of ripening. The concentrations of most amino acids such as serine, glutamate, alanine, L-allothreonine, and methionine then stabilized, whereas those of phenylalanine, ornithine, tyrosine, and lysine continued to increase until the final stage of the ripening. One possible interpretation of this result is that the most significant proteolysis usually occurred in the first 5 months of dry-cured ham maturation (Grossi et al., 2014). Rapid lipolysis of dry-cured ham also occurred during early maturation (Motilva et al., 1993). As shown in Figure 2, the levels of myristic acid, palmitoleic acid, heptadecanoic acid, arachidonic acid, and arachidic acid increased sharply during this period. As the second most abundant SDM in Panxian ham, fatty acids were also closely related to the flavor of dry-cured ham (Shi et al., 2019), and hexanal from the oxidation of n-6 fatty acids like arachidonic acid was an odor-active compound in Istrian and Iberian hams (Marušić et al., 2014).

Glucose, fructose, glycerol, and myo-inositol were identified as the major carbon compounds in Panxian ham. Among them, the concentrations of glucose and fructose declined continually throughout the process, while those of myo-inositol and glycerol increased steadily. Glucose and fructose are often directly associated with sweetness; however, some studies have reported that sugars had little contribution to the taste of dry-cured ham due to its low content in the final product (Zhang et al., 2018). Glycerol and myo-inositol also had a slight effect on perceived sweetness (Seo et al., 2016). Glycerol was a product of fat degradation. Therefore, the increase in glycerol content was linked to lipolytic enzyme activity (Koutina et al., 2012). Myo-inositol was a vitamin-like essential nutrient. Yang et al. (2018) found that higher concentrations of myo-inositol could improve digestion and growth.

Organic acids were also important taste-related compounds in foods. Succinic acid has been identified as a taste-active compound in boneless dry-cured ham (Zhang et al., 2019), and the combination of succinic, lactic, and citric acids can generate unique sour and umami tastes (Dashdorj et al., 2015). Malate was a mild acid and contributed to the red coloration and protein degradation of cured meat (Kim et al., 2019). Sugimoto et al. (2017) found that dry-cured ham with a longer ripening period had a much higher malate content, this corroborated with the present study (Figure 2). Creatine was linked to bitter taste and has been described as a taste-active compound in duck meat (Liu et al., 2013). In general, a decrease in creatine during the initial stage of dry-cured ham ripening was accompanied by an increase in creatinine (Mora et al., 2010). Therefore, the creatinine/creatine ratio was considered useful in estimating the minimum ripening time. However, Escudero et al. (2011) found that the hypoxanthine/inosine ratio was more suitable as a potential biomarker for the primary ripening of dry-cured ham. In addition, the high levels of hypoxanthine can not only enhance the cured meat taste (Ichimura et al., 2017), but also contribute to the taste of boneless dry-cured hams (Zhang et al., 2019).

### Abundance and Diversity of Bacterial and Fungal Microorganisms

**FIGURE 3:**
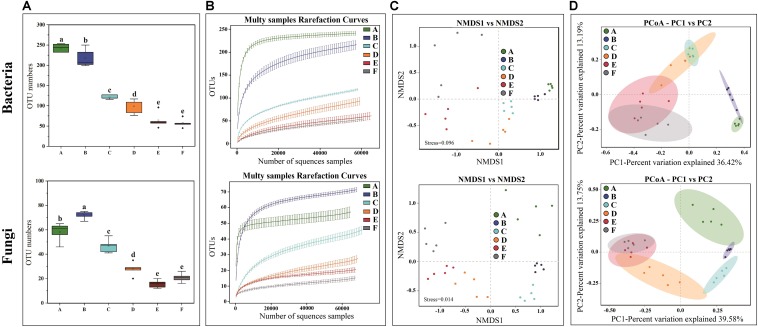
Effects of spontaneous fermentation on microbial diversity and community structure of Panxian ham. **(A)** OTU number of bacteria and fungi at different processing stages, values are presented as mean ± standard error (*n* = 5), ^a–*e*^ different letters represent significant differences (*p* < 0.05). **(B)** rarefaction curves of bacteria and fungi for each sample. **(C,D)** NMDS and PCoA score plots of bacteria and fungi. A–F indicate the traditional spontaneous fermentation process stage of Panxian ham: *(A)* raw ham; *(B)* post-salting; *(C)* post-resting; *(D)* initial stage of ripening; *(E)* middle stage of ripening; *(F)* final stage of ripening.

High-throughput sequencing generated a total of 1,991,084 V3-V4 16S rRNA regions and 2,158,470 ITS1 high-quality sequence reads from 30 Panxian ham samples. The numbers of effective sequences ranged from 62,788 to 71,736 for bacteria and from 68,114 to 75,068 for fungi (Supplementary Table S2). As shown in Figure 3A, the OTU numbers of bacteria and fungi decreased continually during Panxian ham processing, and the numbers of bacteria always higher than that of fungi. The rarefaction curve of each sample based on the OTU number was used to evaluate sequencing depth, which showed that sequencing data was sufficient for subsequent analyses (Figure 3B). Dynamic changes to α-diversity indices indicated that the fungal diversity of Panxian ham fluctuated greatly during processing, while bacterial diversity tended to continually decrease (Table 1). Similar trends have been reported during the Suan yu, a fermentation process for Chinese traditional fermented fish; Zang et al. (2018) suggested that chitin (a characteristic component of the fungal cell wall) may be responsible for these results, providing a certain protection against environmental stress. Microbiota structural changes were analyzed using NMDS and PCoA based on Binary-Jaccard distances to evaluate variation and similarity (Figures 3C,D). The results demonstrated that the succession of bacteria and fungi was not synchronous during Panxian ham processing.

**TABLE 1:** Richness and diversity of bacteria and fungi at different processing stages.

**Samples**	**Richness index**				**Diversity index**			
		
	**ACE**		**Chao 1**		**Simpson**		**Shannon**	
				
	**Bacteria**	**Fungi**	**Bacteria**	**Fungi**	**Bacteria**	**Fungi**	**Bacteria**	**Fungi**
A	245.78 ± 9.80^a^	95.04 ± 30.85^a^	247.17 ± 11.76^a^	68.85 ± 19.92^ab^	0.05 ± 0.01^a^	0.35 ± 0.05^a^	3.90 ± 0.12^a^	1.90 ± 0.22^a^
B	234.68 ± 19.92^a^	77.17 ± 8.24^ab^	231.69 ± 22.70^a^	77.92 ± 6.68^a^	0.14 ± 0.01^a^	0.25 ± 0.02^a^	2.77 ± 0.08^b^	1.83 ± 0.07^a^
C	174.88 ± 37.08^b^	58.18 ± 8.80^bc^	174.24 ± 22.70^b^	58.84 ± 8.36^b^	0.30 ± 0.04^b^	0.73 ± 0.05^c^	1.69 ± 0.10^c^	0.55 ± 0.07^d^
D	195.64 ± 39.90^b^	42.22 ± 18.10^cd^	148.79 ± 25.31^b^	33.70 ± 10.20^c^	0.56 ± 0.18^c^	0.53 ± 0.13^b^	1.03 ± 0.38^d^	0.84 ± 0.23^c^
E	103.57 ± 27.33^c^	22.55 ± 6.39^d^	106.37 ± 47.71^c^	18.45 ± 4.19^c^	0.55 ± 0.11^c^	0.34 ± 0.04^a^	1.02 ± 0.20^d^	1.22 ± 0.12^b^
F	98.82 ± 22.58^c^	24.62 ± 6.24^d^	81.81 ± 11.84^c^	21.89 ± 4.23^c^	0.48 ± 0.12^c^	0.59 ± 0.06^b^	0.79 ± 0.20^d^	0.77 ± 0.13^c^

*Values are presented as mean ± standard error (*n* = 5). A–F indicate the traditional spontaneous fermentation process stage of Panxian ham: (A) raw ham; (B) post-salting; (C) post-resting; (D) initial stage of ripening; (E) middle stage of ripening; (F) final stage of ripening. ^*a*–*d*^Different letters in the same column represent significant differences (*p* < 0.05).*

### Bacterial and Fungal Community Succession During Panxian Ham Processing

**FIGURE 4:**
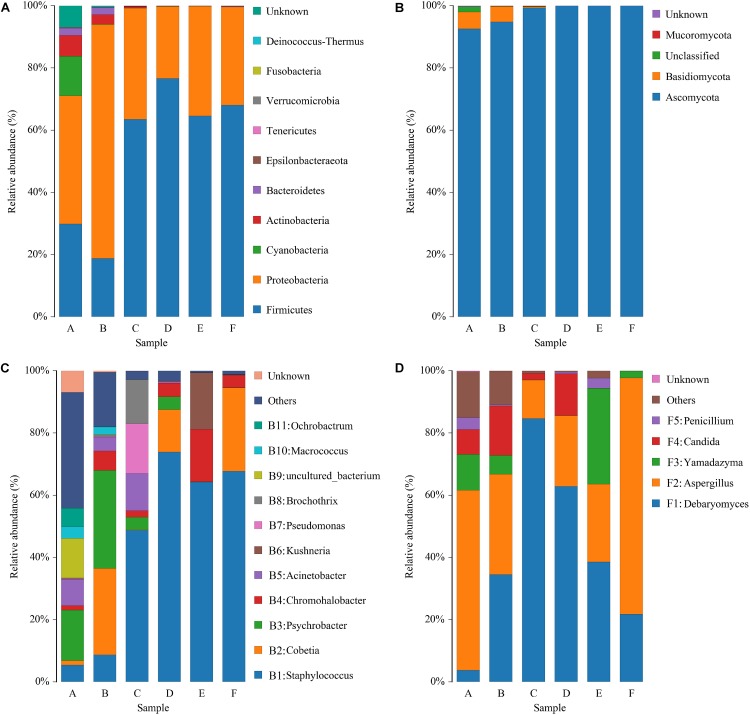
Relative abundance of bacteria **(A)** and fungi **(B)** at the phylum level and bacteria **(C)** and fungi **(D)** at the genus level in samples of Panxian ham. A–F indicate the traditional spontaneous fermentation process stage of Panxian ham: *(A)* raw ham; *(B)* post-salting; *(C)* post-resting; *(D)* initial stage of ripening; *(E)* middle stage of ripening; *(F)* final stage of ripening.

The sequencing data of bacteria and fungi were classified at both the phylum and genus levels to better understand community succession. Ten bacterial phyla were identified; *Firmicutes* and *Proteobacteria* were predominant in all samples (Figure 4A). *Proteobacteria* was the most abundant phylum in raw and post-salting hams, while *Firmicutes* dominated the resting and ripening stages. With respect to fungi, four phyla were identified, and *Ascomycota* represented more than 92% of the sequences of each sample (Figure 4B). Furthermore, *Cyanobacteria*, *Actinobacteria*, *Bacteroidetes*, and *Basidiomycota*, primarily as a result of their presence in raw ham materials, were commonly identified in all early stage ham samples.

At the genus level, 154 bacterial genera were identified during Panxian ham processing and 11 core genera (whose relative abundance was larger than 1%) are shown in Figure 4C. The raw hams were characterized by *Psychrobacter* (16.28%), followed by uncultured_bacterium (12.70%), *Acinetobacter* (8.36%), *Ochrobactrum* (5.98%), *Staphylococcus* (5.44%), and *Macrococcus* (3.79%). After salting, the relative abundance of *Psychrobacter* was further increased to 31.57%, while the relative abundances of uncultured_bacterium and *Ochrobactrum* were remarkably reduced to 0.12% and 0.08%, respectively. Interestingly, the proportion of *Cobetia*, which was reportedly derived from salt (Jung et al., 2015), increased dramatically from an initial value of 1.38 to 27.76%. *Staphylococcus* then became the dominant genus during the resting and ripening stages, with a relative abundance ranging from 48.74 to 73.87%. Similarly, some researchers have reported that *Staphylococcus* was dominant in other fermented meat products (Wang et al., 2018; Gassem, 2019). Coagulase-negative *staphylococci* (CNS) has been regarded as the most important factor in the taste and flavor development of dry-cured ham, after endogenous enzymes (Landeta et al., 2007), owing to its strong enzyme activity, which includes nitrate reductase, catalase, and lipolytic and proteolytic enzymes (Landeta et al., 2013). In addition, CNS also contributed to the preservation of meat products through nitric oxide synthase (Sapp et al., 2014). For other core genera, the relative abundance of *Chromohalobacter* and *Kushneria* increased rapidly at the final stage of ripening. This variation might be related to the increase in salinity, because most members of these two genera were salt-tolerant and were often isolated from high salt foods such as cured meat (Zou and Wang, 2010) and salt-fermented fish (Yun et al., 2018).

Some 51 fungal genera were detected and only five taxa, *Debaryomyces*, *Aspergillus*, *Yamadazyma*, *Candida*, and *Penicillium*, were identified as core fungal genera (Figure 4D). Two dominant groups were present in all samples, *Debaryomyces* and *Aspergillus*, and exhibited obvious antagonism during the whole processing of Panxian ham, which was mainly due to wide nutritional competition between yeasts and molds (Adel Druvefors and Schnurer, 2005). Yeast could promote the sensory characteristics of dry-cured meat products due to exogenous enzyme activities (Dura et al., 2004). As one of the most commonly isolated yeasts from dry-cured ham, *Debaryomyces* often dominated the yeast population after salting (Núñez et al., 1996). *Debaryomyces* inoculation has been reported to promote the development of more volatile compounds related to the flavor of dry-cured meat products (Andrade et al., 2009). Thus, *Debaryomyces* has been widely used as a starter culture to enhance the flavor and aroma of dry-cured meat products (Laranjo et al., 2019). *Aspergillus* was one of the most abundant molds in dry-cured ham. High-quality molds can not only improve the typical features of fermented meat products, but also inhibit the growth of harmful microorganism (Bruna et al., 2003; Castellari et al., 2010). However, Wang et al. (2006) suggested that the production of some undesirable mycotoxins by molds such as *Aspergillus* and *Penicillium* may not contribute to Xuanwei ham quality. Rojas et al. (1991) and Comi and Iacumin (2013) analyzed the toxigenic potential of molds on the surface of Spanish and San Daniele dry-cured ham and concluded that their presence did not pose a health hazard. Meanwhile, the growth of autochthonous yeasts in dry-cured ham has been shown to inhibit the accumulation of ochratoxin A, a significant mycotoxin in processed meats (Simoncini et al., 2014).

### Multivariate Statistical Analysis of Metabolites and Microorganisms

It is generally accepted that variance in the microbial community structure will affect the volatile and non-volatile metabolites present in fermented food that determine its sensory properties, which inevitably affects the final product’s industrial value. This is especially true for foods prepared by spontaneous fermentation such as dry-cured ham. Therefore, multivariate RDA was applied to reveal the correlations between Panxian ham samples, core microbial genera, and SDMs.

**FIGURE 5:**
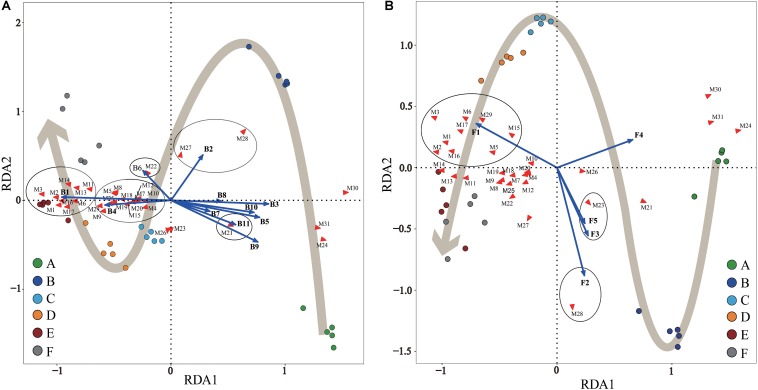
Redundancy analysis (RDA) of Panxian ham samples, core microbial genera, and significantly different metabolites (SDMs) during Panxian ham processing. **(A)** Correlation between samples, core bacteria genera, and SDMs. **(B)** Correlation between samples, core fungi genera, and SDMs. A–F indicate the traditional spontaneous fermentation process stage of Panxian ham: *(A)* raw ham; *(B)* post-salting; *(C)* post-resting; *(D)* initial stage of ripening; *(E)* middle stage of ripening; *(F)* final stage of ripening. The core bacterial and fungal genera were consistent with Figures 4C,D; while SDMs are represented by M1–M31. M1, valine; M2, Isoleucine; M3, proline; M4, glycine; M5, serine; M6, L-allothreonine; M7, aspartic acid; M8, methionine; M9, pyroglutamate; M10, glutamic acid; M11, phenylalanine; M12, ornithine; M13, lysine; M14, tyrosine; M15, alanine; M16, myristic acid; M17, palmitoleic acid; M18, heptadecanoic acid; M19, arachidonic acid; M20, arachidic acid; M21, stearic acid; M22, succinic acid; M23, malic acid; M24, creatine; M25, glycerol; M26, myo-inositol; M27, fructose; M28, glucose; M29, hypoxanthine; M30, inosine; M31, creatinine.

Statistical RDA triplot analysis (Figure 5) demonstrated that the changes in SDMs during Panxian ham processing were the result of interactions between bacterial and fungal communities, but seemed to be dominated by the bacterial population, particularly, *Staphylococcus* and *Chromohalobacter*. These two genera were significantly correlated with the production of all amino acids and fatty acids except stearic acid (Figure 5A). Moreover, *Debaryomyces* was also associated with the metabolism of six amino acids and two fatty acids (Figure 5B). This correlation could be attributed to microbial enzymes originated from these microorganisms. Of these, the proteolytic and lipolytic activities of *Staphylococcus* and *Debaryomyces* were well known (Dura et al., 2004; Landeta et al., 2013). The ability of *Chromohalobacter* to produce industrially important enzymes, including proteases and lipases, had also been previously reported (Kumar et al., 2012). Furthermore, the fermentation of ganjiang, a Korean traditional fermented soy sauce, was dominated by *Chromohalobacter*, which was conducive to the generation of amino and organic acids (Jung et al., 2015). *Aspergillus* and *Cobetia* had a significant and positive correlation coefficient with glucose and fructose. These findings concur with those reported by other authors who noticed that *Aspergillus* was primarily responsible for sugar metabolism in fermented foods (Lee et al., 2017; Tang et al., 2019). However, Jung et al. (2015) reported that *Cobetia* was not associated with the metabolites of ganjiang, although the early stage fermentation samples were represented by *Cobetia*. In addition, *Kushneria*, *Yamadazyma*, and *Penicillium* were closely related with organic acids, namely, succinic and malic acids. It has been reported that some *Penicillium* strains isolated on the surface of dry-cured sausages have malate dehydrogenase activity (Vila et al., 2019), which is crucial to the reversible conversion between malate and oxaloacetate. However, microbial community succession did not account for changes in inositol, creatine, creatinine, and inosine concentrations. Seo et al. (2016) suggested that myo-inositol was not technically a fermentation product and was mainly derived from *Aspergillus* fungus in koji. In addition, the conversion of creatine to creatinine during dry-cured ham processing was dependent on the processing time and initial creatine concentration (Mora et al., 2010).

It is worth noting that *Acinetobacter*, *Psychrobacter*, *Brochothrix*, *Pseudomonas*, *Ochrobactrum*, *Macrococcus*, *Candida*, and uncultured_bacterium showed significant and negative correlation coefficients with most SDMs (Figure 5); these microorganisms represented the microbial community in the early stages of Panxian ham processing (Figure 4). Usually, the incidence and/or prevalence of gram-negative bacteria in fermented meat products is undesirable. *Acinetobacter*, *Psychrobacter*, *Brochothrix*, and *Pseudomonas* were recognized as meat spoilage factors due to their production of off-flavors, and were a hazardous source of transmissible antibiotic resistance (Wang et al., 2018). Additionally, *Pseudomonas* has been proven to be related to the production of biogenic amines in fermented foods (Huang et al., 2018). *Ochrobactrum* is considered an opportunistic bacterium with antimicrobial resistance that has been associated with human wound infections (Furlan and Stehling, 2017). This genus was frequently isolated from soil samples and it was also recently detected in traditional Icelandic fermented fish called hakarl (Osimani et al., 2019). *Macrococcus*, a gram-positive bacterium closely related to *Staphylococcus*, was often found in animal skin samples and their meat products (Sae et al., 2010). Previous researchers concluded that *Macrococcus* has nothing to do with human or animal disease (Lory, 2014), but a strain of *Macrococcus* causing high mortality has been separated from commercial chickens in China (Li et al., 2018). Meanwhile, *Macrococcus* was also one of the main microorganisms responsible for chicken spoilage (Arthur et al., 2004). *Candida* was the most abundant yeast in the fresh ham (Núñez et al., 1996), but it was also recognized as a pathogenic yeast isolated from dry-cured hams (Levenson et al., 1991). Some pathogenic strains of *Candida* can cause both superficial and systemic infections (Carolus et al., 2019). Fortunately, *Staphylococcus*, *Debaryomyces*, *Aspergillus*, *Kushneria*, and *Chromohalobacter* showed strong antagonistic behavior toward these undesirable microorganisms (Supplementary Figure S3). As a result, the relative abundance of these potentially risky microorganisms was reduced and eventually disappeared through traditional spontaneous fermentation (Figure 4), indicating the safety of the final Panxian ham product. The results of gene function prediction from the PICRUSt also supported this viewpoint (Supplementary Figure S4). The abundance of predicted functional genes involved in human diseases decreased significantly during Panxian ham processing. Nevertheless, it is necessary to find measures to reduce the incidence and prevalence of these undesirable microorganisms in the early stages of processing.

## Conclusion

Investigating the dynamic changes in microbial community and metabolite profiles provided information regarding the metabolically active microbiota that promote the production of primary metabolites during spontaneous fermentation of Panxian ham. The results showed that eight microbial genera, namely, *Staphylococcus*, *Chromohalobacter*, *Cobetia*, *Kushneria*, *Debaryomyces*, *Aspergillus*, *Yamadazyma*, and *Penicillium*, were significantly correlated with changes in SDMs. The prevalence of these microbial genera was crucial to the refinement of the sensory qualities and safety of Panxian ham by inhibiting the growth of some pathogen-related microorganisms. These findings play a vital role in understanding Panxian ham quality parameter development; allowing the production of safe, high quality products. Nevertheless, corresponding this type of microbial analysis with sensory parameters (meat color, pH, water loss, texture), volatiles flavor, and taste evaluation by a sensory panel would be highly relevant. Further, metagenomics and transcriptomics should be applied to elucidate the functions of these microorganisms.

## Data Availability Statement

All sequencing data have been deposited at the Sequence Read Archive of the National Center for Biotechnology Information (NCBI), with SRA accession number: the accession number of bacteria and fungi are PRJNA579779 and PRJNA579784, respectively.

## Author Contributions

YM and WS contributed to the experimental design. YM performed the statistical analysis and wrote the manuscript. YM, WS, YCM, and LJ contributed to manuscript revision, and read and approved the submitted version.

## Conflict of Interest

The authors declare that the research was conducted in the absence of any commercial or financial relationships that could be construed as a potential conflict of interest.

## Footnotes

1 www.metaboanalyst.ca

2 www.genome.ad.jp/kegg
