# Supplementary material for: Combined Application of High-Throughput Sequencing and Metabolomics Reveals Metabolically Active Microorganisms During Panxian Ham Processing
Source: Front Microbiol. 2020 Jan 10;10:3012. doi: 10.3389/fmicb.2019.03012 (PMC6966718; doi:10.3389/fmicb.2019.03012)
Supplement: Supplementary file 1 [file Data_Sheet_1.docx]

Supplementary Material

# Supplementary Figures and Tables

## Supplementary Figures


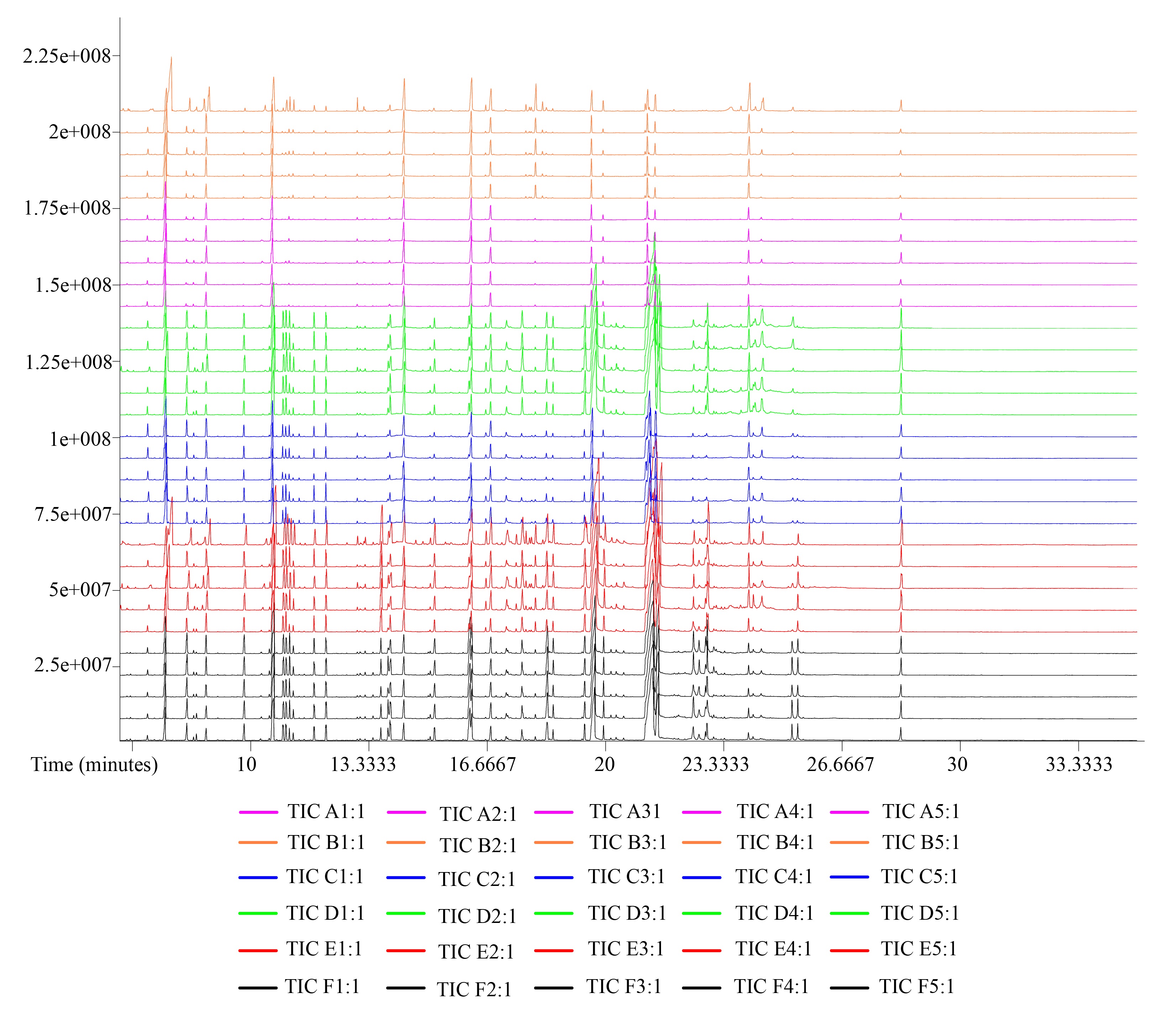


**Figure S1.** The GC-TOF-MS typical total ion chromatograms (TICs) of dry-cured ham samples from different processing stages. A-F indicate the traditional spontaneous fermentation process stage of Panxian ham: A) raw ham; B) post-salting; C) post-resting; D) initial stage of ripening; E) middle stage of ripening; F) final stage of ripening.


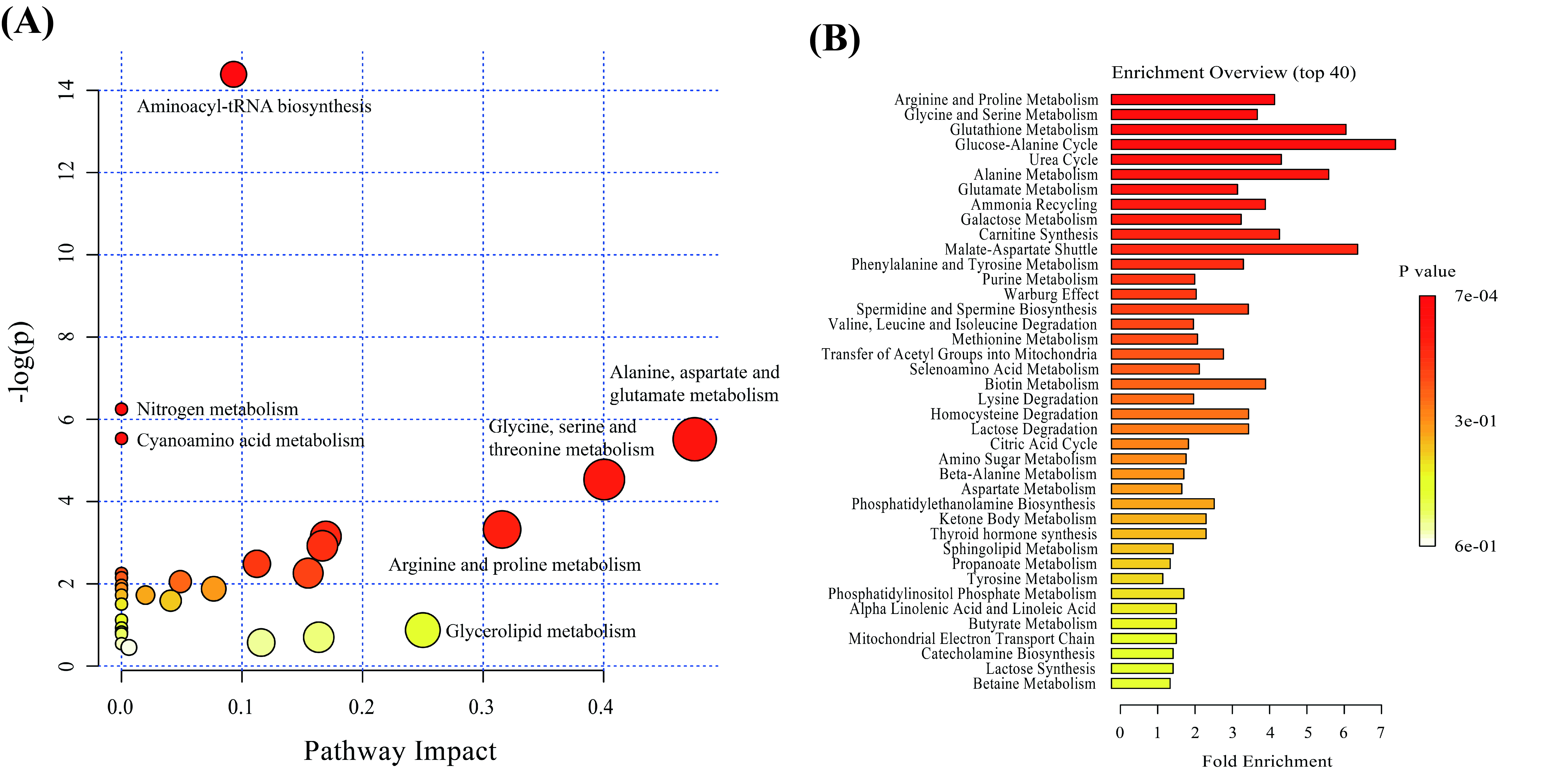


**Figure S2.** Metabolic pathway bubble chart (A) and enrichment analysis histogram graph (B) from MetaboAnalyst.


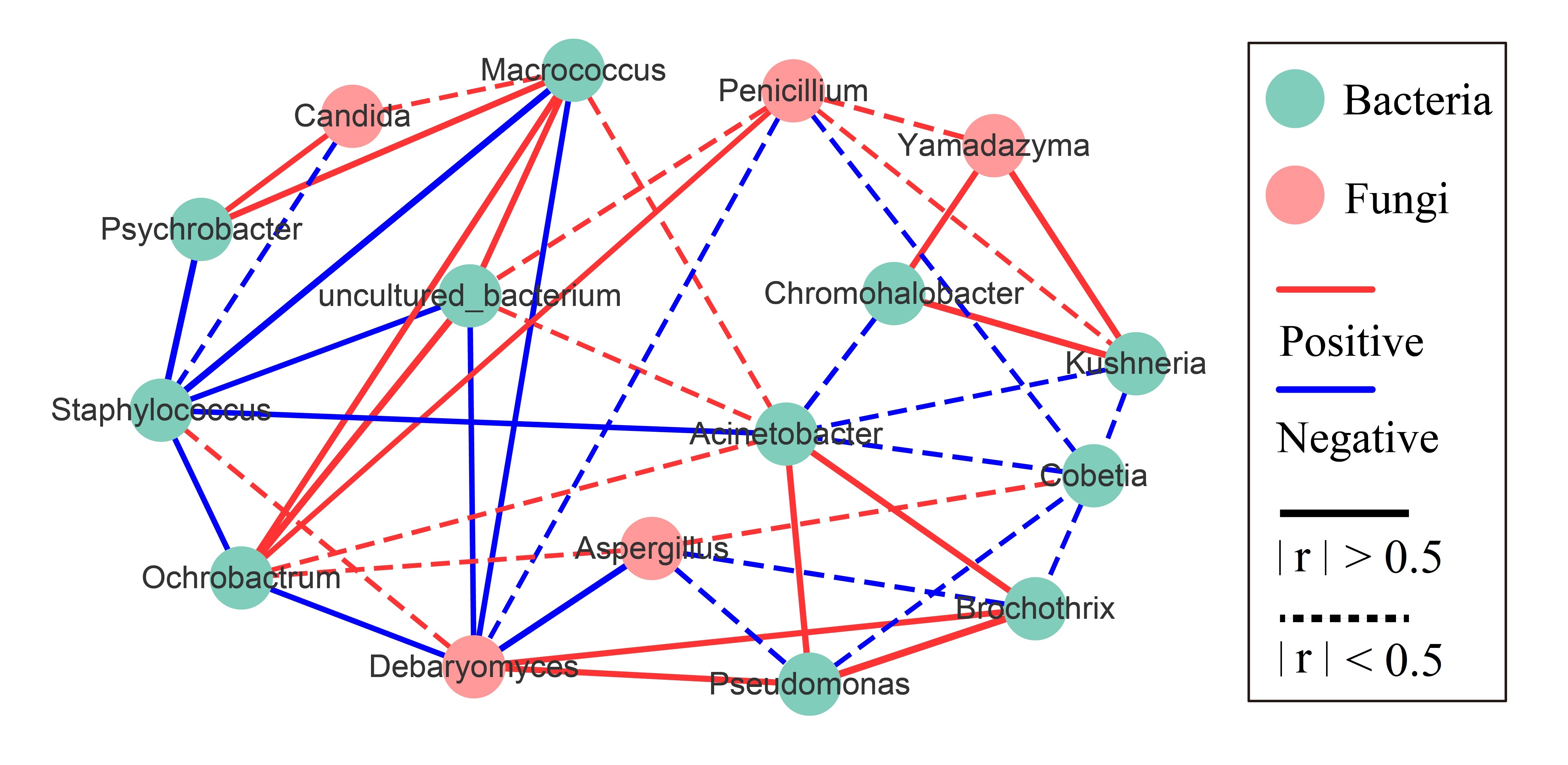


**Figure S3.** Visualization of the co-occurrence network between core bacteria and fungi based on Pearson's correlation coefficient. Only significant edges are drawn in the network (p < 0.05).


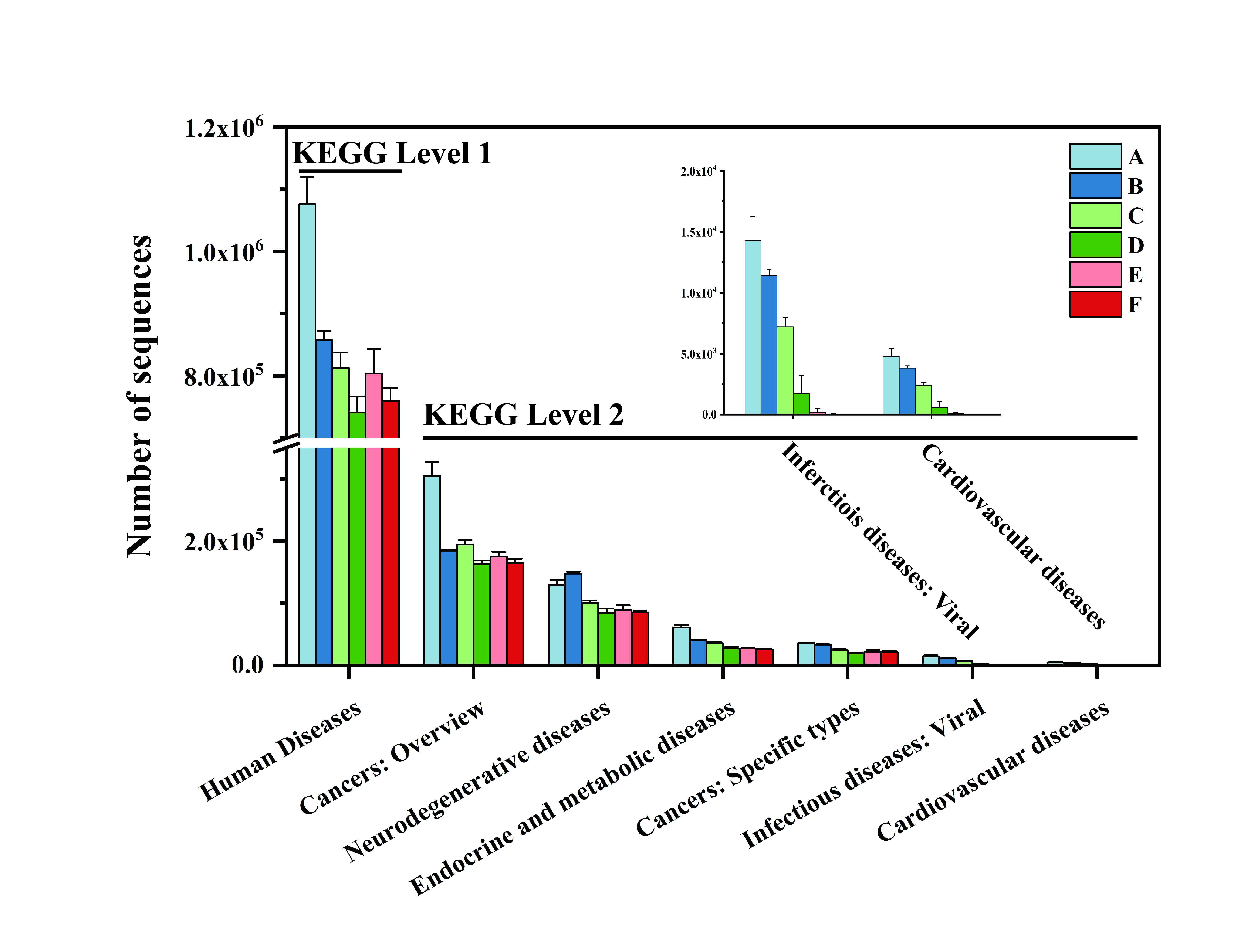


**Figure S4.** Changes in the predicted functional genes associated with human disease by PICRUSt. A-F indicate the traditional spontaneous fermentation process stage of Panxian ham: A) raw ham; B) post-salting; C) post-resting; D) initial stage of ripening; E) middle stage of ripening; F) final stage of ripening.

## Supplementary Tables

**Table S1.** Main variables contributing to discrimination between Panxian ham samples.

| NO. ^a^ | Metabolites | RT (min) | Similarly | CAS No. | Formula | Identified ion (m/z) | VIP | P |
| --- | --- | --- | --- | --- | --- | --- | --- | --- |
| 1 | Alanine | 8.21 | 938.00 | 56-41-7 | C_3_H_7_NO_2_ | 116 | 4.49196 | <0.001 |
| 2 | Valine | 9.81 | 970.57 | 72-18-4 | C_5_H_11_NO_2_ | 144 | 3.51072 | <0.001 |
| 3 | Creatinine | 10.61 | 819.74 | 60-27-5 | C_4_H_7_N_3_O | 115 | 3.57523 | <0.001 |
| 4 | Isoleucine | 10.91 | 933.44 | 7004-09-3 | C_6_H_13_NO_2_ | 158 | 3.22182 | <0.001 |
| 5 | Proline | 10.99 | 922.80 | 344-25-2 | C_5_H_9_NO_2_ | 142 | 3.71771 | <0.001 |
| 6 | Glycine | 11.09 | 972.22 | 56-40-6 | C_2_H_5_NO_2_ | 174 | 2.40088 | <0.001 |
| 7 | Succinic acid | 11.20 | 958.39 | 110-15-6 | C_4_H_6_O_4_ | 147 | 2.52926 | 0.001 |
| 8 | Serine | 11.78 | 911.53 | 56-45-1 | C_3_H_7_NO_3_ | 204 | 2.12469 | <0.001 |
| 9 | Glycerol | 11.81 | 794.44 | 56-81-5 | C_3_H_8_O_3_ | 73 | 3.38433 | <0.001 |
| 10 | L-Allothreonine | 12.12 | 928.17 | 28954-12-3 | C_4_H_9_NO_3_ | 73 | 4.41000 | <0.001 |
| 11 | Malic acid | 13.44 | 911.11 | 97-67-6 | C_4_H_6_O_5_ | 73 | 2.01511 | <0.001 |
| 12 | Aspartate | 13.86 | 908.60 | 56-84-8 | C_4_H_7_NO_4_ | 232 | 1.56552 | <0.001 |
| 13 | Methionine | 13.87 | 910.31 | 63-68-3 | C_5_H_11_NO_2_S | 176 | 1.92582 | <0.001 |
| 14 | Pyroglutamate | 13.93 | 955.17 | 98-79-3 | C_5_H_7_NO_3_ | 156 | 3.88777 | <0.001 |
| 15 | Creatine | 14.32 | 955.89 | 57-00-1 | C_4_H_9_N_3_O_2_ | 115 | 4.52149 | <0.001 |
| 16 | Glutamate | 15.06 | 920.97 | 56-86-0 | C_5_H_9_NO_4_ | 246 | 1.15166 | <0.001 |
| 17 | Phenylalanine | 15.18 | 938.06 | 63-91-2 | C_9_H_11_NO_2_ | 218 | 2.29505 | <0.001 |
| 18 | Hypoxanthine | 17.20 | 936.69 | 68-94-0 | C_5_H_4_N_4_O | 265 | 2.96164 | <0.001 |
| 19 | Ornithine | 17.24 | 847.65 | 70-26-8 | C_5_H_12_N_2_O_2_ | 142 | 1.18102 | <0.001 |
| 20 | Myristic acid | 17.64 | 934.28 | 544-63-8 | C_14_H_28_O_2_ | 117 | 3.54138 | <0.001 |
| 21 | Fructose | 17.75 | 947.83 | 57-48-7 | C_6_H_12_O_6_ | 103 | 2.15359 | <0.001 |
| 22 | Glucose | 18.02 | 911.06 | 50-99-7 | C_6_H_12_O_6_ | 205 | 5.66217 | <0.001 |
| 23 | Lysine | 18.34 | 851.08 | 56-87-1 | C_6_H_14_N_2_O_2_ | 174 | 2.32794 | <0.001 |
| 24 | Tyrosine | 18.52 | 944.11 | 60-18-4 | C_9_H_11_NO_3_ | 218 | 2.81360 | <0.001 |
| 25 | Palmitoleic acid | 19.41 | 943.64 | 373-49-9 | C_16_H_30_O_2_ | 174 | 2.80011 | <0.001 |
| 26 | Myo-inositol | 19.94 | 951.67 | 87-89-8 | C_6_H_12_O_6_ | 218 | 3.72728 | <0.001 |
| 27 | Heptadecanoic acid | 20.51 | 896.31 | 506-12-7 | C_17_H_34_O_2_ | 117 | 1.51145 | <0.001 |
| 28 | Stearic acid | 21.47 | 897.54 | 0057/11/4 | C_18_H_36_O_2_ | 341 | 2.44538 | 0.012 |
| 29 | Arachidonic acid | 22.47 | 841.53 | 506-32-1 | C_20_H_32_O_2_ | 80 | 1.66521 | <0.001 |
| 30 | Arachidic acid | 23.05 | 848.42 | 506-30-9 | C_20_H_40_O_2_ | 117 | 1.30698 | <0.001 |
| 31 | Inosine | 24.04 | 931.34 | 58-63-9 | C_10_H_12_N_4_O_5_ | 73 | 5.62373 | <0.001 |

^a^ Numbered as in the retention time.

**Table S2.** Sequence abundance and length of bacteria and fungi in each Panxian ham sample.

| Sample ID ^a^ | Bacteria | | | |  | Fungi | | | |
| --- | --- | --- | --- | --- | --- | --- | --- | --- | --- |
|  | Raw Reads | Effective Tags | Effective (%) | Average  Length(bp) |  | Raw Reads | Effective Tags | Effective (%) | Average  Length(bp) |
| A1 | 79910 | 65234 | 81.634 | 419 |  | 80040 | 71554 | 89.398 | 249 |
| A2 | 80027 | 66293 | 82.838 | 416 |  | 79647 | 68114 | 85.520 | 231 |
| A3 | 79650 | 66643 | 83.670 | 419 |  | 79868 | 70329 | 88.057 | 229 |
| A4 | 80069 | 64202 | 80.183 | 417 |  | 80419 | 70747 | 87.973 | 231 |
| A5 | 80038 | 67232 | 84.000 | 419 |  | 80088 | 71454 | 89.219 | 232 |
| B1 | 80280 | 64337 | 80.141 | 428 |  | 79771 | 71258 | 89.328 | 250 |
| B2 | 80221 | 62788 | 78.269 | 428 |  | 79881 | 72114 | 90.277 | 251 |
| B3 | 80152 | 63552 | 79.289 | 428 |  | 80076 | 72129 | 90.076 | 250 |
| B4 | 79664 | 63793 | 80.078 | 428 |  | 80023 | 73408 | 91.734 | 256 |
| B5 | 80033 | 63263 | 79.046 | 428 |  | 79858 | 74061 | 92.741 | 251 |
| C1 | 80054 | 65306 | 81.577 | 429 |  | 79779 | 74286 | 93.115 | 266 |
| C2 | 79939 | 63710 | 79.698 | 429 |  | 79845 | 74067 | 92.764 | 269 |
| C3 | 80148 | 65555 | 81.792 | 429 |  | 79922 | 74652 | 93.406 | 265 |
| C4 | 79950 | 65638 | 82.099 | 429 |  | 79848 | 75068 | 94.014 | 265 |
| C5 | 79594 | 65207 | 81.925 | 429 |  | 80206 | 74846 | 93.317 | 264 |
| D1 | 79791 | 66126 | 82.874 | 429 |  | 79893 | 71581 | 89.596 | 267 |
| D2 | 79905 | 64401 | 80.597 | 429 |  | 79971 | 71251 | 89.096 | 247 |
| D3 | 79922 | 68751 | 86.023 | 429 |  | 80097 | 72228 | 90.176 | 262 |
| D4 | 79938 | 68922 | 86.219 | 429 |  | 80080 | 71512 | 89.301 | 263 |
| D5 | 79819 | 69221 | 86.722 | 429 |  | 79985 | 71462 | 89.344 | 261 |
| E1 | 79553 | 71468 | 89.837 | 429 |  | 80067 | 71273 | 89.017 | 250 |
| E2 | 80395 | 68341 | 85.007 | 429 |  | 80377 | 71450 | 88.894 | 256 |
| E3 | 80090 | 68740 | 85.828 | 429 |  | 79915 | 72066 | 90.178 | 264 |
| E4 | 79993 | 69727 | 87.166 | 429 |  | 80051 | 70961 | 88.645 | 268 |
| E5 | 79726 | 70219 | 88.075 | 429 |  | 79984 | 70376 | 87.988 | 256 |
| F1 | 79852 | 64992 | 81.391 | 429 |  | 80116 | 71937 | 89.791 | 229 |
| F2 | 80015 | 71736 | 89.653 | 429 |  | 79793 | 70790 | 88.717 | 235 |
| F3 | 79937 | 66609 | 83.327 | 429 |  | 79862 | 71018 | 88.926 | 236 |
| F4 | 79877 | 65065 | 81.456 | 429 |  | 80089 | 71566 | 89.358 | 229 |
| F5 | 80033 | 64013 | 79.983 | 429 |  | 80121 | 70912 | 88.506 | 232 |

^a^ A-F indicate the traditional spontaneous fermentation process stage of Panxian ham: A) raw ham; B) post-salting; C) post-resting; D) initial stage of ripening; E) middle stage of ripening; F) final stage of ripening.
